# Using implementation mapping to refine strategies to improve implementation of an evidence-based mobile market intervention: a study protocol

**DOI:** 10.3389/frhs.2024.1288160

**Published:** 2024-02-13

**Authors:** Christina M. Kasprzak, Andy Canizares, Anne Lally, Jill N. Tirabassi, Leah N. Vermont, Samuel Lev, Alice S. Ammerman, Lucia A. Leone

**Affiliations:** ^1^ Department of Community Health and Health Behavior, School of Public Health and Health Professions, University at Buffalo, Buffalo, NY, United States; ^2^ Department of Anthropology, College of Arts and Sciences, University at Buffalo, Buffalo, NY, United States; ^3^ Department of Family Medicine, Jacobs School of Medicine and Biomedical Sciences, University at Buffalo, Buffalo, NY, United States; ^4^ Department of Nutrition, Gillings School of Global Public Health, University of North Carolina at Chapel Hill, Chapel Hill, NC, United States

**Keywords:** implementation mapping, implementation strategies, dietary intake, mobile produce market, fruits and vegetables, food access

## Abstract

**Objectives:**

The Veggie Van model is a mobile market model that is efficacious in increasing fruit and vegetable consumption for lower-income participants. The model is currently being evaluated for its effectiveness in a multi-state trial. Preliminary implementation data, collected through process measures surveys and implementation interviews, indicate that there are several barriers to implementation among partner organizations and implementation fidelity to the Veggie Van model was low. Consideration and planning for implementation ought to occur early and often throughout the research process order to ensure Veggie Van model effectiveness. This paper describes the step-by-step process for creating strategies to enhance implementation of Veggie Van model components.

**Methods:**

Implementation mapping is a systematic process to develop implementation strategies through engagement with key stakeholders. We conducted a series of interviews (*n* = 31 representatives) with partner organizations (*n* = 8) to identify facilitators and barriers to Veggie Van model implementation. We then applied interview findings to an Implementation Mapping process to develop theory and practice-driven strategies to be integrated into existing implementation tools and technical assistance.

**Results:**

We identified implementation outcomes (e.g., staff implement the Veggie Van model component of nutrition education with fidelity) and performance objectives (e.g., offer nutrition education, in the form of food lessons and/or food demonstrations, at least bi-weekly) to achieve them. We conducted a secondary qualitative analysis of the findings from implementation interviews with partner organizations to identify behavioral determinants (e.g., attitudinal beliefs, social support) which were combined with the performance objectives to generate change objectives (e.g., view the Veggie Van model as advantageous to an organization and communities served). To achieve the change objectives, we developed implementation strategies that would be integrated into existing Veggie Van training resources including an online toolkit, webinars and trainings, an annual mobile market conference, and technical assistance.

**Conclusion:**

The development of theory and practice-driven implementation strategies will enable us to improve our implementation tools, thereby improving fidelity to the Veggie Van model among organizations and increasing the likelihood of its effectiveness. Detailing the design of a multifaceted implementation strategy using Implementation Mapping also provides a model to design similar strategies for other community-based interventions.

## 1 Introduction

**Figure 1:**
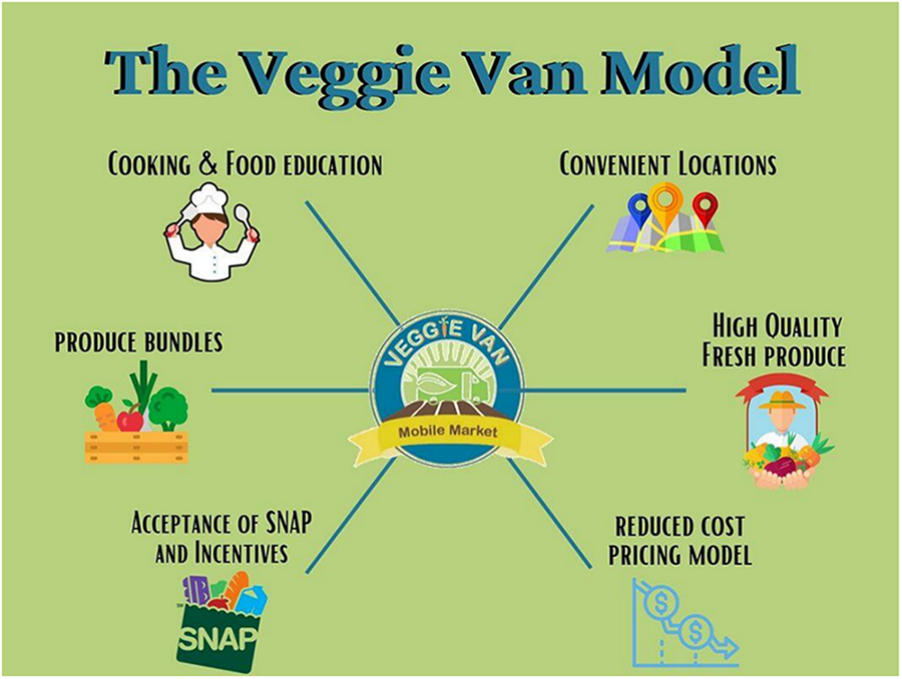
The Veggie Van model components.

Veggie Van is an evidence-based model for mobile produce markets informed by Social Cognitive Theory and designed to address the multiple dimensions of access to fresh fruits and vegetables (F&V) for lower-income and underserved communities: acceptability, availability, affordability, accessibility, and accommodation (1). The Veggie Van intervention (mobile market following. the Veggie Van model) was tested in a 12-site cluster randomized controlled trial and found to be efficacious in increasing F&V consumption for lower-income participants over a 6-month period. Compared to study participants living in control communities, participants in communities that received access to the Veggie Van experienced a .95 cups/day greater change (*p* = 0.005) in F&V consumption at follow-up (2). Intervention participants also reported increases in self-efficacy for preparing and incorporating fresh F&V into meals and snacks (2). Figure 1 illustrates the six core components of the Veggie Van model, which include: (1) operating the mobile market at convenient locations that are selected based on strong partnerships with community-based organizations already working with the intended population of low-income families, (2) operate the mobile market regularly (e.g., weekly visits to the same location), (3) selling reduced cost fresh F&V through a combination of locally competitive pricing and participation in F&V incentive programs, (4) offering a variety of fresh, high quality F&V (e.g., prioritizing local produce), (5) encouraging F&V purchasing by offering and incentivizing bundles of produce (multiple items for a set price), and (6) offering regular nutrition education (e.g., cooking demonstrations, nutrition lessons) (1, 2). Although not a core component of the model, organizations that implement a Veggie Van intervention are encouraged to maintain consistent access for customers by operating at least 10–12 months out of the year.

An ongoing hybrid effectiveness-implementation study seeks to determine if the Veggie Van intervention impact F&V intake and other health-related outcomes in underserved communities when implemented by community organizations in multiple contexts. This 7-year study, known as the Veggie Van Study, also aims to understand how easy or hard it is for organizations to implement the Veggie Van model. Nine study partner organizations across several states in the Eastern U.S. were identified through a request-for-partners (RFP) process; details regarding the recruitment process and effectiveness study methods are reported elsewhere (3, 4). Briefly, partner organizations were provided funding to start or expand mobile market operations following the Veggie Van model for 12 months (4). Partner organizations were asked to partner with community sites serving lower-income areas to help host their mobile market and facilitate recruitment for the study (4). Representatives from each organization had access to a web-based Veggie Van toolkit and regular contact (i.e., email, web conference) with the study team to receive ongoing support for study activities and implementation of the Veggie Van model. Preliminary implementation data, collected through process measures surveys and implementation interviews, indicate that implementation fidelity to the Veggie Van model components among initial study partner organizations is low and there are persistent barriers to implementation (5). For example, although organizations successfully launched or expanded their mobile market operations, partners often did not implement all of the key components of the model (e.g., produce bundles) or at the recommended intensity (e.g., weekly). Therefore, there is a need to develop strategies to enhance implementation of the Veggie Van model components. Implementation strategies are approaches to enhance the adoption and implementation of evidence-based practices (6–8), but available implementation strategies to enhance healthy eating interventions have mainly been employed and tested in school and childcare settings. Furthermore, there is a lack of rigorous and theory-driven design of these strategies (9). Implementation mapping is a form of intervention mapping that guides planners through a systematic process to develop implementation strategies through engagement with key stakeholders (e.g., adopters, implementers) (9, 10). Implementation mapping has been used by several studies, but there is a recognized lack of description, justification, and use of theory in designing implementation strategies for public health interventions (6, 9–11). The goal of this paper is to add to the literature by describing our process of utilizing Implementation Mapping to design new implementation strategies for the Veggie Van intervention. Illustrating the step-by-step process of designing a multifaceted implementation strategy will provide a template for others to similarly design strategies for implementation of community-based evidence-based interventions. Strategies developed from this process will also be used to facilitate implementation of Veggie Van interventions among current and future study partners as well as prepare the model to go to scale and be implemented by practitioners more broadly. These strategies will also be used by the Veggie Van Training Center, a service center based at the University at Buffalo that supports community-based organizations to start mobile produce markets and related food access programs. The Veggie Van Training Center currently employs several implementation methods including the Veggie Van toolkit, a cohort-style course known as Mobile Market 101 (MM101), a webinar series on special topics, ongoing technical assistance, and individualized consulting. Therefore, the strategies developed from the Implementation Mapping process are intended to inform updates to these existing tools.

## 2 Methods

We carried out an Implementation Mapping process to design implementation strategies; our process was modeled after the methods of Fernandez and colleagues (9). Implementation mapping involves five tasks: (1) conduct an implementation needs assessment, (2) identify adoption and implementation outcomes, performance objectives, determinants, and change objectives, (3) select theoretical methods and design implementation strategies, (4) produce implementation protocols and materials, and (5) evaluate implementation outcomes. Each task we conducted is outlined in the results section; briefly, for task one, we conducted implementation interviews with representatives from organizations participating in the Veggie Van effectiveness-implementation study. Task two involved a brainstorm session among key members of the Veggie Van research team. The lead and second authors conducted a literature review and engaged in several brainstorm sessions, with input from the Veggie Van Training Center team, for the third task. Tasks four and five are forthcoming. The interviews conducted as part of task one were approved as exempt research by the University at Buffalo Institutional Review Board Study.

### 2.1 Theoretical framework

The Consolidated Framework for Implementation Research (CFIR) informed both the data collection and analysis processes used to identify contextual factors that impact implementation of the Veggie Van model. CFIR is an implementation determinants framework developed in 2009 by Damschroder and colleagues in response to a call for a greater use of theory to guide implementation research (12, 13). CFIR is comprised of 39 constructs within five major domains that interact to influence implementation of programs and interventions and their eventual effectiveness (12).

## 3 Results

### 3.1 Task 1: Conduct an implementation needs assessment

Qualitative data collected through implementation interviews were used for an implementation needs assessment. Interviews were conducted with representatives from eight Veggie Van study partner organizations who were implementing the Veggie Van intervention. Each organization was asked to identify the individuals who most closely engage in the implementation of Veggie Van to participate in two 90-minute semi-structured interviews. Up to four representatives from each organization were invited to participate in implementation interviews at baseline (shortly before or after market launch) and follow-up (9–12 months post market launch), to understand their experience implementing the Veggie Van intervention.

Seventeen representatives from eight study partner organizations completed baseline implementation interviews and fourteen representatives from six organizations completed follow-up interviews. Due to staff turnover, thirteen individuals were interviewed at both timepoints, and one new staff member was interviewed at follow-up. Table 1 presents the experience level of participating organizations and the number and characteristics of interviewees at baseline and follow-up. Each partner organization received compensation for participating in the larger RCT study. Interviews were completed as a part of interviewees' job function; thus, they did not receive additional compensation. Representatives that participated in a baseline interview but left the partner organization prior to follow-up and were still willing to participate in the follow-up interview were compensated with a $50 gift card for each hour they were interviewed.

**Table 1:** Organization and interviewee characteristics.

Mobile market experience at baseline	Number of organizations	Number of individuals interviewed at baseline	Number of individuals interviewed at follow-up
Not currently running a mobile market; operated a CSA program in the past	*n* = 1	*n* = 4	*n* = 3
Not currently running a mobile market; currently operating a free food distribution program	*n*-2	*n* = 5	*n* = 5
Currently operating a mobile market; less than 2 years of experience	*n* = 3	*n* = 5	*n* = 3
Currently operating a mobile market; 2 or more years of experience	*n* = 2	*n* = 3	*n* = 3
Region represented		Mobile market representative job titles	Number of individuals^a^
Northeastern United States (NY, MA)	*n* = 3	Executive director	*n* = 4
Midwestern United States (OH)	*n* = 2	Coordinator	*n* = 3
Southeastern United States (NC)	*n* = 3	Director	*n* = 4
		Program associate	*n* = 2
		Market manager	*n* = 5

^a^ Represents the 17 interviewees interviewed at baseline and 1 new staff member interviewed at follow-up.

Semi-structured interview guides, informed by CFIR, were used for data collection. Relevant CFIR constructs were selected from the 39 measures in the 5 domains of CFIR (characteristics of intervention, outer setting, inner setting, characteristics of individuals, and process). Identification of the most salient constructs for inclusion in the interview guide was guided by our past research with 21 key informants representing established mobile market organizations across the US; findings from that formative work highlighted persistent operational challenges faced by organizations across the US that may undermine implementation of an evidence-based intervention (e.g., the Veggie Van model) (14). The final interview guides focused on the following CFIR constructs: relative advantage, adaptability, complexity, design quality and packaging, cost, external policy and incentives, customer needs and resources, cosmopolitanism, peer pressure, structural characteristics, implementation climate, readiness for implementation, knowledge and beliefs about the intervention, self-efficacy, planning, engaging, and executing. Both interview guides were designed to understand the factors in each organization that either enhance or impede implementation of Veggie Van model components.

Interviews were recorded and transcribed; transcripts were data cleaned for errors and inconsistencies. Qualitative data analysis was completed using the software ATLAS.ti version 8.0. Baseline and follow-up interview data were coded by two independent coders (CK & AC). Each coder analyzed all interview transcripts separately. The first phase of coding involved an inductive organization of data utilizing open coding to identify barriers and facilitators to implementation (15). A second phase of coding involved a deductive organization of interview data (baseline and follow-up) into the 5 domains of CFIR (e.g., inner setting) (12, 15). The creation and iterative refinement of a codebook throughout the coding process allowed for themes across codes to be combined into inductive subcategories (e.g., funding and resources) (15). Regular meetings between coders involved discussion and updates to the codebook throughout the coding process. Coding of baseline interviews was completed first and the codebook generated from that coding process was utilized for subsequent coding of the follow-up interviews, but continual updating and refinements occurred during the coding of follow-up data. Baseline and follow-up data were coded with the same analytical approach, but we iteratively updated the codebook to reflect additional CFIR constructs that were added to the follow-up interview guide. All coded baseline and follow-up data were ultimately merged into one project bundle and the coders met to reconcile coding differences. Reports and queries were generated for facilitators and barriers, for each CFIR construct, across all partner organizations. Memos were written to summarize each code report and query.

In our analysis, we identified barrier and facilitators to implementation that fall under the following CFIR constructs and sub-constructs: intervention characteristics (subconstructs: relative advantage, adaptability, complexity, design quality and packaging, and cost), outer setting (subconstructs: customer needs and resources, cosmopolitanism, peer pressure, and external policy and incentives), inner setting (subconstructs: structural characteristics, implementation climate, and readiness for implementation), and characteristics of individuals (subconstructs: knowledge and beliefs about the intervention and self-efficacy), and process (subconstructs: planning, engaging, and executing). Facilitators and barriers are presented in Table 2 according to CFIR construct. Our findings indicated that there were perceptions of complexity for implementing the Veggie Van model components, particularly offering produce bundles and extending the season. Perceptions that implementing model components requires more staffing, funding, and organizational capacity were also cited barriers, while advantages such as the Veggie Van model being adaptable and providing an evidence base were identified as facilitators to implementation. Concerns surrounding costs related to implementation was a perceived barrier; conversely, understanding that the Veggie Van model is a more a financially sustainable model than a free food distribution market models was a facilitator. Other barriers included: a perceived conflict between organizational mission and/or community preferences and implementing certain Veggie Van model components (e.g., the Veggie Van interventions is a paid model in contrast with one organization that had been previously implementing a free food distribution model), challenges with navigating external barriers (e.g., restrictive policies, unsupportive partners), lack of planning for implementation, and low or worsening self-efficacy to implement the model and its individual components. Networking with other practitioners implementing the model and specialized training in the Veggie Van model components were identified as facilitators to implementation.

**Table 2:** Facilitators and barriers to Implementation of the veggie Van model.

Deductive sorting (CFIR domains)	Intervention characteristics	Outer setting	Inner setting	Characteristics of individuals	Process
Inductive categories (CFIR constructs) Barriers (B) Facilitators (F)	**Relative advantage:** B: VV model requires more staffing; reduced cost pay model is a disadvantage F: VV model helps with formalization of practices	**Customer needs and resources** B: Community pushback for some VV model components (e.g., reduced cost pay model, bundling, local produce) F: VV model helps to meet community need	**Structural characteristics:** B: Insufficient staff size or composition F: Past mobile market or food aggregation/distribution experience	**Knowledge and beliefs about the intervention:** B: VV model does not wholly address access barriers F: VV model is effective at improving access to healthy food	**Planning:** B: N/A F: Strategic planning with host sites
	**Adaptability:** B: N/A F: VV model is flexible and able to be adapted	**Cosmopolitanism:** B: N/A F: Networking with other mobile market practitioners is beneficial	**Implementation climate:** B: N/A F: Organization is receptive to VV model implementation	**Self-efficacy:** B: Low self-efficacy to implement VV model F: High self-efficacy to implement VV model	**Engaging:** B: Lack of awareness of mobile markets or reluctance to shop at a mobile market F: Community acceptance of mobile markets
	**Complexity of the intervention:** B: Some VV model components (e.g., bundling, season extension) are challenging F: Implement VV model at new market sites	**Peer pressure:** B: N/A F: VV model gives organizations an advantage through an evidence-based framework	**Tension for change:** B: Organizations concerns for meeting VV model recommendations and long-term sustainability F: Organization looking for motivation to launch or improve mobile market programming		**Engaging (Champions):** B: N/A F: Residents and community partners serve as valued champions through outreach and resource sharing
	**Design quality and packaging:** B: VV model expertise is inconsistent F: VV model toolkit is thorough and can be tailored	**External policy and incentives:** B: Regulations (e.g., health code, parking) create barriers F: Strong relationships with local health department help with navigating policy	**Compatibility:** B: Conflict between organizational mission and VV model components (e.g., season extension) F: Alignment between VV model and organizational mission		**Engaging (Stakeholders):** B: Community partners (e.g., host sites) and government are unsupportive or not deeply engaged with community F: Community partners and government are supportive, have deep ties with the community, and share resources
	**Cost of intervention:** B: Increased costs from some VV model components (e.g., bundling, season extension, nutrition education) F: VV model will cover or reduce overall expenses		**Relative priority:** B: Conflict between leading priorities due to sharing staff and resources F: Other leading priorities complement VV model implementation		**Executing:** B: VV model was not implemented according to plan F: VV model was implemented according to plan
			**Access to knowledge and information:** B: VV model training was not timely F: Access to TA from research team		
			**Leadership engagement:** B: Insufficient support from board F: Support from leadership (management, director)		
			**Available resources:** B: Insufficient funding, resources, and time F: More funding and resources would facilitate implementation		

### 3.2 Task 2: Identify adoption and implementation outcomes, performance objectives, determinants, and change objectives

For task two, individuals from the Veggie Van study research team and Veggie Van Training Center that had in-depth knowledge of the Veggie Van model and experience with understanding of mobile market operations participated in a brainstorm session. Drawing on the collective experiences from both research and practice and our findings from the needs assessment (task 1), the planning team drafted adoption outcomes, implementation outcomes, and performance objectives for the implementation of the Veggie Van model. Adoption and implementation outcomes were guided by broader goals of what is considered necessary to achieve prior to implementation (adoption) and during implementation to achieve optimal implementation of the Veggie Van model. Discussion of the specific tasks required to achieve those outcomes guided the selection of performance objectives. Performance objectives answer the question, “*What do program [implementers] need to do to deliver the essential program components*?” (9). Organizations participating in the Veggie Van Study completed monthly process measures surveys for the study period which assess implementation of each of the Veggie Van model components. Preliminary process measures data indicate that fidelity to the model, including the dose or intensity (e.g., frequency of nutrition education lessons), greatly varies between and within organizations for individual model components; therefore, the granular nature of our performance objectives reflects this variability in implementation. Implementation outcomes and performance objectives were subsequently refined through follow-up email correspondence between group members. Table 3 outlines the final implementation outcomes and performance objectives.

**Table 3:** Implementation outcomes and performance objectives.

Target: role	Adoption and implementation outcomes	Performance objectives
Adopters and implementers: mobile market staff member(s) and/or leadership	Staff and/or leadership reflect on implementation of the Veggie Van model and its alignment with their organization and the communities the mobile market serves.	1. Identify how the Veggie Van model components align with organizational mission and community needs tailor components if necessary.
		2. Identify the benefits of the Veggie Van model and advantages of implementing the model.
		3. Set a purpose for implementing the Veggie Van model.
	Staff and/or leadership plan for implementation of the Veggie Van model.	4. Prioritize implementation of the Veggie Van model relative to other programming.
		5. Determine costs associated with implementing the Veggie Van model and an organization's financial means to implement.
		6. Establish goals and benchmarks related to Veggie Van model implementation and monitor progress toward goals and benchmarks.
Implementers: mobile market staff member(s)	Staff implement the Veggie Van model component of nutrition education with fidelity.	7. Offer nutrition education, in the form of food lessons and/or food demonstrations, at least bi-weekly (in-person or virtually).
	Staff implement the Veggie Van model component of bundling with fidelity.	8. Offer at least one produce bundle regularly (at least weekly)
	Staff implement the Veggie Van model component of a reduced cost pay model with fidelity.	9. Implement some form of a reduced cost pay model (e.g., suggested price, sliding scale, set price) with posted prices.
		10. Participate in at least one incentive program that increases affordability of produce.
	Staff implement the Veggie Van model component of procuring high quality produce with fidelity.	11. Establish and implement a procurement plan that prioritizes quality and locally/regionally grown produce.
	Staff implement the Veggie Van model component of convenient location with fidelity.	12. Determine mobile market location through partnerships with organizations providing services to lower-income and/or food insecure communities.
		13. Operate the mobile market at regularly (at least 3 times per month, at least 10 months out of the year).
	Staff overcome barriers to implementation of the Veggie Van model.	14. Utilize training and technical assistance for implementing the Veggie Van model and troubleshooting barriers.
		15. Utilize networks (other mobile markets) to support implementation of the Veggie Van model.

Next, the lead and second authors conducted a secondary qualitative analysis of the findings from the formative work on barriers and facilitators to implementation (needs assessment) to identify determinants. Determinants answer the question, “*Why does an individual choose to, or choose not to, implement Veggie Van model components?*” (9). The use of health behavior theories such as the social cognitive theory and theory of planned behavior is an accepted practice for intervention mapping, including Implementation Mapping (6, 9, 16). These theories are widely used, psychometrically valid, and reliable in predicting and influencing health behaviors (e.g., cigarette smoking, exercise) through individual-level determinants (16). Constructs and determinants from individual-level theories including social cognitive theory, health belief model theory, and theory of planned behavior were incorporated into a codebook (17–19). Memos were then deductively coded by each author to identify individual level determinants among interviewees (implementers) that were relevant to implementation. The authors met to discuss and reconcile codes; final theories (social cognitive theory, theory of planned behavior, and health belief model) were chosen based on the frequency of determinants represented and some determinants were collapsed into overarching concepts. Many of our aforementioned findings corresponded to the constructs perceived benefits and barriers (health belief model), attitudes (theory of planned behavior), and outcome expectations/expectancies (social cognitive theory). We consolidated these constructs into the overarching determinant of “attitudinal beliefs”. Other constructs that are represented by the findings included social support and norms (theory of planned behavior, social cognitive theory), self-efficacy and skills (social cognitive theory), and perceived control (health belief model) (17–19). Next, a matrix of change was created in which performance objectives are combined with the personal determinants in order to produce change objectives. Change objectives are discrete changes that are required for each relevant determinant that will ideally lead to achieving performance objectives (9). Table 4 is the final matrix of change outlining performance objectives, identified determinants, and change objectives.

**Table 4:** Matrix of change objectives for implementation of the veggie Van model.

Performance objectives	Behavior: mobile market staff implementation of the Veggie Van model personal determinants			
	Attitudinal beliefs	Social support and norms^a^	Perceived control	Self-efficacy and skills^a^ (behavioral capability)
PO1. Reconcile organizational mission and community needs with Veggie Van model values; tailor model if necessary.	**AB.1** Perceive the Veggie Van model as complimentary to organizational mission, market model, and community members' needs.		**PC.1** Express control over tailoring the Veggie Van model to align with an organization's values and community members' needs.	
PO2. Recognize benefits of the Veggie Van model and the advantages of implementing the model.	**AB.2** View the Veggie Van model as advantageous to an organization and communities served.	**SS/N.2** Recognize that implementing the Veggie Van model gives an organization and advantage over other organizations due to its evidence base.		
PO3. Establish realistic expectations of outcomes of Veggie Van model.	**AB.3** Expect that the benefits of implementing the Veggie Van model are limited in their scope and cannot wholly address systemic issues (e.g., poverty, health disparities).			
PO4. Prioritize implementation of the Veggie Van model	**AB.4.a** View the implementation of the Veggie Van model as a top priority. **AB.4.b** View the implementation of the Veggie Van model as complimentary to other top priorities.		**PC.4** Express control over prioritizing the implementation of the Veggie Van model.	
PO5. Reflect on costs associated with implementing the Veggie Van model and an organization's means to implement.	**AB.5.a** Expect that implementing the Veggie Van model may increase overall expenses but also financial sustainability. **AB.5.b** View the Veggie Van model as a cost-effective model that enhances financial sustainability.		**PC.5** Express control over implementing the Veggie Van model, despite insufficient funding and/or resources.	
PO6. Establish goals and benchmarks related to Veggie Van model implementation and monitor progress toward goals and benchmarks.				**SE/S.6** Demonstrate the confidence and skills to establish goals and benchmarks for Veggie Van model implementation and monitor progress toward goals.
PO7. Offer nutrition education, in the form of food lessons and/or food demonstrations, at least bi-weekly (in-person or virtually).	**AB.7.a** Expect that conducting regular nutrition education will lead to increased customer self-efficacy and skills to consume F&V. **AB.7.b** Perceive that the benefits to conducting nutrition education outweigh the costs.	**SS/N.7** Connect and exchange ideas with other practitioners to facilitate implementing nutrition education.	**PC.7** Express control over implementing nutrition education (internally and/or through partnerships) and tailoring to meet organizational and community needs.	**SE/S.7** Demonstrate the confidence and skills to implement nutrition education on a regular basis (internally and/or through partnerships).
PO8. Offer at least one produce bundle regularly (at least weekly)	**AB.8.a** Expect that implementing a produce bundle regularly will be beneficial by increasing customer buying power, exposing customers to new types of produce, and facilitate pandemic adaptations. **AB.8.b** Perceive that the benefits to implementing a produce bundle outweigh the barriers.	**SS/N.8.a** Connect and exchange ideas with other practitioners to facilitate implementing produce bundles. **SS/N.8.b** Recognize that other mobile markets are implementing a produce bundle.	**PC.8** Express control over tailoring the produce bundle to meet community members' needs.	**SE/S.8** Demonstrate the confidence and skills to implement a produce bundle regularly.
PO9. Implement some form of a reduced cost pay model (e.g., suggested price, sliding scale, set price) with posted prices.	**AB.9.a** Expect that implementing a reduced cost payment model will be beneficial through increasing affordability, supporting local farmers, and enhancing program sustainability. **AB.9.b** View a reduced cost payment model as integral to increase affordability of F&V while enhancing program sustainability.	**SS/N.9.a** Determine whether implementing a reduced cost payment model is aligned with organizational mission and community expectations. **SS/N.9.b** Recognize that implementing a reduced cost payment model is a common strategy among mobile markets to enhance sustainability.	**PC.9** Express control over tailoring the component of reduced cost payment model to align with community members' needs and organizational mission.	**SE/S.9** Demonstrate the confidence and skills to implement a reduced cost payment model.
PO10. Participate in at least one incentive program that increases affordability of produce.	**AB.10.a** Expect that participating in the SNAP program incentive program(s) will increase affordability and increase overall market sales. **AB.10.b** View SNAP acceptance and participation in incentive program(s) as an integral to increasing affordability of F&V.		**PC.10** Express control over enrolling and participating in the SNAP and incentive program(s) at the mobile market.	**SE/S.10** Demonstrate the confidence and skills to implement SNAP and at least one incentive program.
PO11. Establish and implement a procurement plan that prioritizes quality and locally/regionally grown produce.	**AB.11.a** Expect that prioritizing high quality produce procurement will be beneficial through satisfying customer preference and increasing sales. **AB.11.b** Expect that prioritizing local produce procurement will be beneficial through supporting local agriculture. **AB.11.c** View procurement of high quality, local/regional when possible, produce as a priority.		**PC.11** Express control and flexibility over procuring high quality, with a preference toward local/regional, produce.	**SE/S.11** Demonstrate the confidence and skills to procure high quality, local/regional when possible, produce.
PO12. Determine mobile market location through partnerships with organizations providing services to lower-income and/or food insecure communities.	**AB.12.** Expect that identifying market locations through partnerships will be beneficial through enhancing community reach, community engagement and trust, and the viability of market sites.		**PC.12.a** Express control over positioning the mobile market in an accessible location in chosen communities. **PC.12.b** Express control over establishing expectations and accountability practices with stakeholders (e.g., host sites).	**SE/S.12** Demonstrate the confidence and skills to partner with community organizations to host the mobile market.
PO13. Operate the mobile market at regularly (at least 3 times per month, at least 10 months out of the year).	**AB.13.a** Expect that operating the market regularly will strengthen community engagement and consistently increase healthy food access. **AB.13.b** Perceive that the benefits to operating the mobile market regularly outweigh the barriers.	**SS/N.13.a** Determine whether operating an extended season is aligned with organizational mission. **SS/N.13.b** Recognize that adapting market setup to adjust to the weather (i.e., moving indoors) is commonplace among mobile markets.	**PC.13** Express control over adapting the extended market season to meet organizational and community needs.	**SE/S.13** Demonstrate the confidence and skills to operate the mobile market regularly (at least 3 times per month, at least 10 months out of the year)
PO14. Utilize training and technical assistance for implementing the Veggie Van model and troubleshooting barriers.	**AB.14.a** Expect that utilizing technical assistance will lead to better implementation of the Veggie Van model. **AB.14.b** Perceive that the benefits to utilizing training and technical assistance outweigh the barriers.			
PO15. Utilize networks (other mobile markets) to support implementation of the Veggie Van model.	**AB.15.a** Expect that utilizing networks of mobile market practitioners will lead to better implementation of the Veggie Van model. **AB.15.b** Perceive that the benefits to utilizing mobile market networks outweigh the barriers.	**SS/N.15** Recognize that other mobile markets in the US are implementing the Veggie Van model and collectively have an impact.		

^a^ We have combined these related, but distinct constructs, merely to conserve space.

### 3.3 Task 3: Select theoretical methods and design implementation strategies

A literature review identified taxonomies of theory-based behavior change techniques from Michie et al., Kok et al., and Abraham et al. (20–22). The determinant of perceived control was not represented in these taxonomies, but we identified a textbook on perceived control and behavior change (23). The techniques from this literature were synthesized in a master spreadsheet that outlined each method, definition, and parameters (i.e., conditions necessary for a particular change to be effective); techniques were also organized based on which individual determinant they target. Through discussion among the first and second authors, techniques were primarily chosen based on relevance to the individual determinants that were identified in our needs assessment. For example, the behavior change technique of modeling involves demonstrating a desired action or behavior (e.g., implementing the produce bundle components of the Veggie Van model) and could be employed to target self-efficacy and skills. The choice of behavior change techniques was also guided by practical considerations of what can feasibly be integrated into existing implementation resources and relevance to program implementers. For example, the technique of fear arousal involving presentation of mortality information is not relevant to the context of implementing the Veggie Van model. Furthermore, providing normative information about others' behavior (implementation of the Veggie Van model) is more feasible to incorporate into existing tools compared to mass media role modeling which involves enforcing norms through mass media.

The chosen behavior change techniques informed the design of more tangible implementation strategies. Implementation strategies were drafted by the lead and second author and were finalized after receiving feedback from the planning team. For example, one strategy for the online toolkit is to include instruction on how to implement the Veggie Van model component of a produce bundle which is intended to increase self-efficacy and behavioral capability. Determinants are targeted through multiple methods (i.e., toolkit, training) and many of the strategies target multiple determinants. Table 5 presents the final implementation plan with chosen behavior change methods and corresponding individual determinants that are targeted.

**Table 5:** Implementation plan for supporting Implementation of the veggie Van model.

Implementation strategies [change objectives addressed]	Channels and vehicles	Determinants	Behavior change methods
Update content and curriculum to contain messaging that strengthens positive attitudes toward the Veggie Van model, weakens negative attitudes toward the Veggie Van model, and is tailored to the viewer and their community. **[Change Objectives: AB.1, AB.2, AB.4.a, AB.4.b, AB.5.b, AB.7.b, AB.8.b, AB.9.b, AB.10.b, AB.11.c, AB.13.b]**	• Website (Veggie Van toolkit) • Meetings (webinars/trainings, summit, MM101)	Attitudinal beliefs (attitudes, outcome expectations/expectancies, perceived benefits/perceived barriers)	Elaboration^a^ Belief selection^a^
Update content and curriculum to contain messaging that strengthens positive expectations of the Veggie Van model, weakens negative expectations of the Veggie Van model, and is tailored to the viewer and their community. **[Change Objectives: AB.3, AB.5.a, AB.7.a, AB.8.a, AB.9.a, AB.10.a, AB.11.a, AB.11.b, AB.12, AB.13.a]**	• Website (Veggie Van toolkit) • Meetings (webinars/trainings, summit, MM101)	Attitudinal beliefs (attitudes, outcome expectations/expectancies, perceived benefits/perceived barriers)	Elaboration^a^ Belief selection^a^
Update content to contain messaging that strengthens positive attitudes toward technical assistance (TA) and networking, weakens negative attitudes toward TA and networking, and is tailored to the viewer and their community. **[Change Objectives: AB.14.b, AB.15.b]**	• Website (Veggie Van Toolkit)	Attitudinal beliefs (attitudes, outcome expectations/expectancies, perceived benefits/perceived barriers)	Elaboration^a^ Belief selection^a^
Update content and curriculum to contain messaging that strengthens positive expectations of TA and networking, weakens negative expectations of TA and networking, and is and is tailored to the viewer and their community. **[Change Objective: AB.15.a, AB.14.a]**	• Website (Veggie Van toolkit) • Meetings (webinars/trainings, summit, MM101)	Attitudinal beliefs (attitudes, outcome expectations/expectancies, perceived benefits/perceived barriers)	Elaboration^a^ Belief selection^a^
Update content to contain checklists and worksheets to guide organizations through establishing implementation goals, planning their actions toward achieving goals, and monitoring progress toward goals. **[Change Objectives: SE/S.6]**	• Website (Veggie Van toolkit)	Self-efficacy and skills (behavioral capability)	Action planning^b^ Goal setting^a^^,^^b^^,^^c^ Self-monitoring of behavior^a^^,^^b^^,^^c^
Update content and TA practices to include instruction and modeling on how to implement each of the Veggie Van model components. **[Change Objetives: SE/S.7, SE/S.8, SE/S.9, SE/S.10, SE/S.11, SE/S.12, SE/S.13]**	• Website (Veggie Van toolkit) • TA and consulting	Self-efficacy and skills (behavioral capability)	Provide instruction on how to perform the behavior^b^^,^^c^ Modeling^a^^,^^b^^,^^c^
TA engagements to include praise/rewards that are linked to implementing each of the Veggie Van model components. **[Change Objetives: SE/S.8, SE/S.9, SE/S.10, SE/S.11, SE/S.12, SE/S.13]**	• TA and consulting	Self-efficacy and skills (behavioral capability)	Provide contingent rewards^a^^,^^b^^,^^c^
Update content and curriculum to contain messaging that describes the prevalence of Veggie Van model implementation among US mobile market organizations. **[Change Objective: SS/N.15]**	• Website (Veggie Van toolkit) • Meetings (webinars/trainings, summit, MM101)	Social support and norms	Provide normative information about others' behavior^b^
Update content to contain messaging that describes the prevalence of US mobile markets that are implementing the more complex Veggie Van model components. **[Change Objectives: SS/N.8.b, SS/N.9.b, SS/N.13.b]**	• Website (Veggie Van toolkit)	Social support and norms	Provide normative information about others' behavior^b^
Update content to contain testimonials from mobile market organizations implementing the Veggie Van model to highlight the advantages of the model. **[Change Objectives: SS/N.2]**	• Website (Veggie Van toolkit)	Social support and norms	Provide normative information about others' behavior^b^
Host events that encourage social networks and cultivate linkages (buddy system, mentoring) among organizations implementing the Veggie Van model to provide support to each other for implementing the more complex Veggie Van model components. **[Change Objectives: SS/N.8.a, SS/N.7]**	• Meetings (webinars/trainings, summit, MM101)	Social Support and Norms	Developing new network linkages^a^ Mobilizing social networks^a^
Integrate motivational interviewing (MI)-informed language into TA engagements to strengthen positive attitudes toward the Veggie Van model and weaken negative attitudes toward the Veggie Van model. **[Change Objectives: AB.1, AB.2, AB.3, AB.4.a, AB.4.b, AB.5b]**	• TA and consulting	Attitudinal beliefs (attitudes, outcome expectations/expectancies, perceived benefits/perceived barriers)	MI^a^^,^^b^^,^^c^
Integrate MI-informed language into TA engagements to promote a sense of control over implementing the Veggie Van model. **[Change Objectives: PC.4, PC.5, PC.7, PC.8, PC.9, PC.10, PC.11, PC.12.a, PC.12.b, PC.13]**	• TA and consulting	Perceived control	MI^a^^,^^b^^,^^c^ Cognitive restructuring^d^
Integrate MI-informed language into TA engagements to determine if implementing certain Veggie Van model components is aligned with organizational mission and community expectations (norms). **[Change Objectives: SS/N.13.a, SS/N.9.a]**	• TA and Consulting	Social Support and Norms	MI^a^^,^^b^^,^^c^
TA engagements include working with organizations to anticipate and/or identify financial barriers associated with implementing the Veggie Van model and identify ways to overcome them. **[Change Objectives: AB.5.b]**	• TA and consulting	Attitudinal beliefs (attitudes, outcome expectations/expectancies, perceived benefits/perceived barriers)	Barrier identification/problem solving^a^^,^^b^^,^^c^
TA engagements include working with organizations to anticipate and/or identify barriers associated with implementing each of the Veggie Van model components and identify ways to overcome them. **[Change Objectives: AB.7.b, AB.8.b, AB.9.b, AB.10.b, AB.11.c, AB.13.b]**	• TA and consulting	Attitudinal beliefs (attitudes, outcome expectations/expectancies, perceived benefits/perceived barriers)	Barrier identification/problem solving^a^^,^^b^^,^^c^
TA engagements include working with organizations to anticipate and/or identify barriers associated with utilizing networks of mobile market practitioners that are similarly implementing the Veggie Van model and identify ways to overcome them. **[Change Objectives: AB.15.b]**	• TA and consulting	Attitudinal beliefs (attitudes, outcome expectations/expectancies, perceived benefits/perceived barriers)	Barrier identification/problem solving^a^^,^^b^^,^^c^
TA engagements include guiding organizations through establishing implementation goals, planning their actions toward achieving goals, and monitoring progress toward goals. **[Change Objectives: SE/S.6]**	• TA and consulting	Self-efficacy and skills (behavioral capability)	Action planning^b^ Goal setting^a^^,^^b^^,^^c^ Self-monitoring of behavior^a^^,^^b^^,^^c^

^a^ Behavior change methods was derived from Kok et al. (20).

^b^ Behavior change methods was derived from Michie et al. (21).

^c^ Behavior change methods was derived from Abraham et al. (22).

^d^ Behavior change methods was derived from Robinson et al. (23).

## 4 Discussion

This research contributes to the field of implementation science through demonstrating our process of using Implementation Mapping to design strategies that can enhance implementation of a food access intervention known as the Veggie Van Model. To our knowledge, this is the first published Implementation Mapping process for a community-based intervention addressing food access and F&V consumption. Implementation Mapping has been conducted overwhelmingly for evidence-based interventions intended for clinical settings; the few examples of Implementation Mapping for community-based interventions have been in fields such as rehabilitation science (24), cancer prevention (25), and physical activity (26). Needs assessments from these Implementation Mapping processes involved quantitative data collection or informal discussion (24, 25); though, one process similarly conducted extensive formative work identifying facilitators and barriers to implementation (26). A strength of this research is that we conducted our needs assessment with 31 representatives from organizations that were actively implementing the Veggie Van model across eight cities that were having mixed success with implementation. The scope of our needs assessment provided a breadth of data with rich detail on facilitators and barriers of implementing the model across unique organizations and communities. Another strength of this research is the usage of the implementation framework CFIR, that has been recommended for the Implementation Mapping process (9). The benefit of utilizing CFIR is that it organized our qualitative findings in a way that allowed us to fully understand the many dimensions of implementation. For example, the benefits of networking among practitioners (CFIR construct: cosmopolitanism) and its impact on implementation of the Veggie Van model were not immediately apparent to our research team until we conducted interviews that were guided by this framework. This research is unique in that implementation tools and resources (i.e., TA, toolkit) were already in place which allowed us to focus on how to augment these existing resources through refining the content and messaging conveyed through these channels. We hope that this approach inspires others to systematically improve their implementation resources in non-clinical settings.

Our implementation interviews (step 1: needs assessment) yielded important factors that help or hinder implementation at levels of the socioecological model beyond the individual. As such, we recognize that even well designed and executed strategies that target the individual implementer may still be limited by these organizational, community, and policy-level constraints. Many of these challenges further validate our past formative work identifying common operational challenges among mobile market organizations (14) and justify the need for further exploration of the interplay between individual and environmental factors. Technical assistance is an effective strategy aimed at building organizational capacity to support implementation (27, 28) and could be viewed as a way of helping organizations overcome barriers at multiple levels including the environment. However, there is opportunity to identify additional strategies to more directly influence the outer spheres of the socioecological model to improve implementation.

This research is not without its limitations. Utilizing active study partner organizations for our needs assessment may bias our findings because these organizations are likely more committed to implementing the Veggie Van model given that it was a requirement of participation in a research study and have greater means to implement due to receiving research funding and technical assistance. However, evaluating implementation among highly invested partners has forged strong relationships between the research team and mobile market organizations which in turn facilitates in-depth and long-term implementation data collection. Conducting future implementation interviews with organizations that are not implementing the Veggie Van model as a requirement of an intervention study would provide context on implementing the model with less support. Finally, a more community-engaged approach could have been employed in this process which could have involved inviting representatives outside of our research lab, namely those that participated in interviews, to contribute to the design of these strategies.

We are currently conducting tasks four and five of the Implementation Mapping process. Task four entails updating all of our implementation tools and protocols based on the strategies outlined here. We have created an implementation intervention protocol for the Veggie Van Training Center, fidelity checklists for technical assistance engagements, and have begun making content edits to the online toolkit. Task five will involve evaluating the impact of our strategies on implementation of the Veggie Van model through interviews and monthly process measures surveys with new cohorts of organizations enrolled in MM101. Outcomes of interest will include adoption and fidelity to the Veggie Van model but also psychosocial determinants. Despite the common use of behavior change techniques in Implementation Mapping, it is unclear whether the application of these techniques and purported mechanisms of action (i.e., individual determinants and constructs) are applicable to implementing behaviors given that the evidence base is rooted in health intentions and behaviors. We hope to help elucidate the role of these determinants among implementers over time and in relation to our implementation strategies through this impending evaluation work. Finally, interviews were mostly conducted with implementers, rather than adopters, and the interview guide was focused on implementation of the Veggie Van model. We recognize that adoption is a distinct but related process to implementation, and we hope to expand our understanding of the factors that influence the decision to initially adopt the Veggie Van model and the progression from adoption to implementation in future research.

## 5 Conclusion

There is a dearth of evidence-based implementation strategies to enhance the implementation of community-based interventions. Researchers and practitioners that are interested in improving fidelity to similar food access interventions, particularly those with existing implementation tools in place, may benefit from modeling after our process.

## Data Availability

The raw data supporting the conclusions of this article will be made available by the authors, without undue reservation. This trial is registered under clinicaltrials.gov (NCT04246593). Registered on January 29, 2020.

## References

[1] Leone LA Tripicchio GL Haynes-Maslow L McGuirt J Grady Smith JS Armstrong-Brown J A cluster-randomized trial of a mobile produce market program in 12 communities in North Carolina: program development, methods, and baseline characteristics. J Acad Nutr Diet. (2019) 119(1):57–68. 10.1016/j.jand.2018.04.010 29945851 PMC6309644

[2] Leone LA Tripicchio GL Haynes-Maslow L McGuirt J Grady Smith JS Armstrong-Brown J Cluster randomized controlled trial of a mobile market intervention to increase fruit and vegetable intake among adults in lower-income communities in North Carolina. Int J Behav Nutr Phys Act. (2018) 15(1):2. 10.1186/s12966-017-0637-1 29304862 PMC5756418

[3] Leone LA Kasprzak C Lally A Haynes-Maslow L Vermont LN Horrigan-Maurer C A novel process to recruit and select community partners for a hybrid implementation-effectiveness study. Prog Community Health Partnersh. (2023) 17(1):159–71. 10.1353/cpr.2023.0021 37462585 PMC10569409

[4] Vermont LN Kasprzak C Lally A Claudio A Tumiel-Berhalter L Haynes-Maslow L Ammerman A A randomized controlled trial of a research-tested mobile produce market model designed to improve diet in under-resourced communities: rationale and design for the veggie van study. Int J Environ Res Public Health. (2022) 19(16):9832. 10.3390/ijerph19169832 36011468 PMC9408281

[5] Kasprzak CM. Evaluation of Pre-implementation Capacity and Implementation Effectiveness of an Evidence-Based-Intervention for Mobile Produce Markets (Ph.D.). State University of New York at Buffalo, United States, New York (2022).

[6] Proctor EK Powell BJ McMillen JC. Implementation strategies: recommendations for specifying and reporting. Implement Sci. (2013) 8:139. 10.1186/1748-5908-8-139 24289295 PMC3882890

[7] Powell BJ Fernandez ME Williams NJ Aarons GA Beidas RS Lewis CC Enhancing the impact of implementation strategies in healthcare: a research agenda. Front Public Health. (2019) 7:3. 10.3389/fpubh.2019.00003 30723713 PMC6350272

[8] Powell BJ McMillen JC Proctor EK Carpenter CR Griffey RT Bunger AC A compilation of strategies for implementing clinical innovations in health and mental health. Med Care Res Rev. (2012) 69(2):123–57. 10.1177/1077558711430690 22203646 PMC3524416

[9] Fernandez ME Ten Hoor GA van Lieshout S Rodriguez SA Beidas RS Parcel G Implementation mapping: using intervention mapping to develop implementation strategies. Front Public Health. (2019) 7:158. 10.3389/fpubh.2019.00158 31275915 PMC6592155

[10] Fernandez ME Ruiter RAC Markham CM Kok G. Intervention mapping: theory- and evidence-based health promotion program planning: perspective and examples. Front Public Health. (2019) 7:209. 10.3389/fpubh.2019.00209 PMC6702459 31475126

[11] Davies P Walker AE Grimshaw JM. A systematic review of the use of theory in the design of guideline dissemination and implementation strategies and interpretation of the results of rigorous evaluations. Implement Sci. (2010) 5(1):14. 10.1186/1748-5908-5-14 20181130 PMC2832624

[12] Damschroder LJ Aron DC Keith RE Kirsh SR Alexander JA Lowery JC. Fostering implementation of health services research findings into practice: a consolidated framework for advancing implementation science. Implement Sci. (2009) 4:50. 10.1186/1748-5908-4-50 19664226 PMC2736161

[13] Kirk MA Kelley C Yankey N Birken SA Abadie B Damschroder L. A systematic review of the use of the consolidated framework for implementation research. Implement Sci. (2016) 11:72. 10.1186/s13012-016-0437-z 27189233 PMC4869309

[14] Kasprzak CM Lally A Schoonover JJ Gallicchio DR Haynes-Maslow L Vermont LN Operational challenges that may affect implementation of evidence-based mobile market interventions. BMC Public Health. (2022) 22(1):776. 10.1186/s12889-022-13207-8 35429973 PMC9013179

[15] Elo S Kyngas H. The qualitative content analysis process. J Adv Nurs. (2008) 62(1):107–15. 10.1111/j.1365-2648.2007.04569.x 18352969

[16] Bartholomew Eldredge LK Markham CM Ruiter RAC Fernández ME Kok G Parcel GS. Planning Health Promotion Programs: An Intervention Mapping Approach. Hoboken: John Wiley & Sons (2016).

[17] Bandura A. Health promotion by social cognitive means. Health Educ Behav. (2004) 31(2):143–64. 10.1177/1090198104263660 15090118

[18] Ajzen I. The theory of planned behaviour: reactions and reflections. Psychol Health. (2011) 26(9):1113–27. 10.1080/08870446.2011.613995 21929476

[19] Rosenstock IM Strecher VJ Becker MH. Social learning theory and the health belief model. Health Educ Q. (1988) 15(2):175–83. 10.1177/109019818801500203 3378902

[20] Michie S Ashford S Sniehotta FF Dombrowski SU Bishop A French DP. A refined taxonomy of behaviour change techniques to help people change their physical activity and healthy eating behaviours: the CALO-RE taxonomy. Psychol Health. (2011) 26(11):1479–98. 10.1080/08870446.2010.540664 21678185

[21] Kok G Gottlieb NH Peters GJ Mullen PD Parcel GS Ruiter RA A taxonomy of behaviour change methods: an intervention mapping approach. Health Psychol Rev. (2016) 10(3):297–312. 10.1080/17437199.2015.1077155 26262912 PMC4975080

[22] Abraham C Michie S. A taxonomy of behavior change techniques used in interventions. Health Psychol. (2008) 27(3):379–87. 10.1037/0278-6133.27.3.379 18624603

[23] Robinson SA Lachman ME. Perceived control and behavior change: a personalized approach. In: Reich JW Infurna FJ, editors. Perceived Control: Theory, Research, and Practice in the First 50 Years. New York: Oxford University Press (2016). p. 0.

[24] Kang E Foster ER. Use of implementation mapping with community-based participatory research: development of implementation strategies of a new goal setting and goal management intervention system. Front Public Health. (2022) 10:834473. 10.3389/fpubh.2022.834473 35619816 PMC9127132

[25] Ibekwe LN Walker TJ Ebunlomo E Ricks KB Prasad S Savas LS Using implementation mapping to develop implementation strategies for the delivery of a cancer prevention and control phone navigation program: a collaboration with 2-1-1. Health Promot Pract. (2020) 23(1):86–97. 10.1177/1524839920957979 33034213 PMC8032810

[26] Walker TJ Kohl HW Bartholomew JB Green C Fernández ME. Using implementation mapping to develop and test an implementation strategy for active learning to promote physical activity in children: a feasibility study using a hybrid type 2 design. Implement Sci Commun. (2022) 3(1):26. 10.1186/s43058-022-00271-9 35256018 PMC8899444

[27] Scott VC Jillani Z Malpert A Kolodny-Goetz J Wandersman A. A scoping review of the evaluation and effectiveness of technical assistance. Implement Sci Commun. (2022) 3(1):70. 10.1186/s43058-022-00314-1 35765107 PMC9238031

[28] Katz J Wandersman A. Technical assistance to enhance prevention capacity: a research synthesis of the evidence base. Prev Sci. (2016) 17(4):417–28. 10.1007/s11121-016-0636-5 26858179 PMC4839040

